# Assessment of the Effect of Rapid Maxillary Expansion on Nasal Respiratory Function and Obstructive Sleep Apnea Syndrome in Children: A Systematic Review

**DOI:** 10.3390/jcm14186565

**Published:** 2025-09-18

**Authors:** Alessio Danilo Inchingolo, Grazia Marinelli, Mirka Cavino, Lucia Pia Zaminga, Sara Savastano, Francesco Inchingolo, Gianluca Martino Tartaglia, Massimo Del Fabbro, Andrea Palermo, Angelo Michele Inchingolo, Gianna Dipalma

**Affiliations:** 1 Department of Interdisciplinary Medicine, University of Bari “Aldo Moro”, 70121 Bari, Italy; alessiodanilo.inchingolo@uniba.it (A.D.I.); grazia.marinelli@uniba.it (G.M.); mirka.cavino@uniba.it (M.C.); luciapia.zaminga@uniba.it (L.P.Z.); sarasavastano@uniba.it (S.S.); francesco.inchingolo@uniba.it (F.I.); angelomichele.inchingolo@uniba.it (A.M.I.); gianna.dipalma@uniba.it (G.D.); 2 Department of Biomedical, Surgical and Dental Science, Milan University, 20122 Milan, Italy; gianluca.tartaglia@unimi.it (G.M.T.); massimo.delfabbro@unimi.it (M.D.F.); 3 Unit of Maxillo-Facial Surgery and Dentistry, Fondazione IRCCS Ca’ Granda Ospedale Maggiore Policlinico, 20122 Milan, Italy; 4 Department of Experiment Medicine, University of Salento, 73100 Lecce, Italy

**Keywords:** nasal breathing, rapid expansion, maxillary, OSAS

## Abstract

**Background**: Obstructive sleep apnea syndrome (OSAS) and impaired nasal breathing are common in children and are frequently linked to maxillary constriction. Rapid maxillary expansion (RME) is an orthopedic treatment used to increase upper airway dimensions and improve respiratory function. It has been hypothesized that RME could contribute to improvements in behavior and cognition, possibly through enhanced sleep and respiratory function. It also promotes the shift from oral to nasal breathing, supporting craniofacial development and neuromuscular stability, and it is increasingly recognized as a multidisciplinary intervention that can improve pediatric health outcomes. With increasing evidence supporting its efficacy, RME should be considered not only for its orthodontic benefits but also as a multidisciplinary treatment option within pediatric care protocols. This underscores the importance of integrated care among orthodontists, ENT specialists, and pediatricians. **Aim:** To systematically assess the impact of RME on nasal respiratory parameters and sleep-disordered breathing, particularly OSAS, in pediatric patients. **Methods:** Following PRISMA guidelines, a systematic review was conducted using 12 clinical studies evaluating anatomical and functional respiratory changes after RME in children with mouth breathing or OSAS. Parameters included airway volume (CBCT, cephalometry), nasal resistance (rhinomanometry), and polysomnography (PSG) data. **Results:** RME consistently resulted in significant increases in nasal cavity volume and upper airway dimensions. Multiple studies reported reductions in the apnea–hypopnea index (AHI), improved oxygen saturation, and better subjective sleep quality. Longitudinal studies confirmed the stability of these benefits. However, variability in study protocols limited meta-analytical comparison. **Conclusions:** RME is effective in enhancing nasal breathing and mitigating OSAS symptoms in children. While results are promising, further high-quality randomized controlled trials are needed to validate these findings and guide standardized treatment protocols.

## 1. Introduction

### 1.1. Rapid Maxillary Expansion (RME)

Rapid maxillary expansion (RME) is a well-established orthopedic–orthodontic procedure primarily employed during the developmental stages of childhood and adolescence to address transverse maxillary deficiencies [1,2,3,4,5].

**Figure 1:**
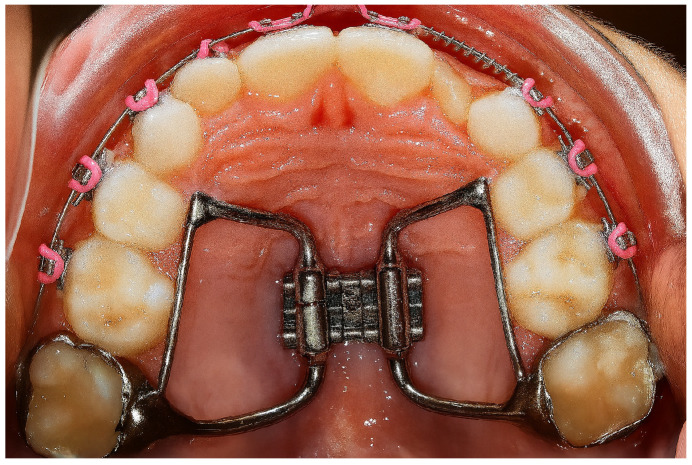
Rapid maxillary expansion.

The RME technique consists of the progressive activation of a specific expansion device (Figure 1), which can be anchored to the dentition (tooth-borne devices) or to the basal bone of the maxilla (bone-borne devices). This device applies transverse orthopedic forces capable of opening the midpalatal suture and promoting skeletal widening of the upper arch [6,7,8,9,10].

Depending on the type of anchorage and activation protocol, the expansion may be rapid or semi-rapid, and it results in a permanent increase in the transverse dimensions of both the dental arch and the skeletal base.

The primary objective of RME is to normalize interarch relationships by expanding the maxillary arch to achieve a better fit with the mandible, thereby restoring occlusal harmony and fostering balanced craniofacial development during growth [11,12,13,14,15,16,17,18,19].

In growing individuals, the midpalatal suture is still responsive to mechanical stimuli, which enhances the skeletal effects of RME and increases its therapeutic efficacy.

Importantly, the effects of RME are not confined to the dentoalveolar and skeletal domains [20,21,22,23,24,25,26,27,28,29,30,31]. By increasing the transverse dimension of the palate, RME simultaneously induces anatomical and functional changes in adjacent structures, especially the nasal cavity and the floor of the nasal fossae [32,33,34,35,36,37,38,39]. The expansion of the nasal base reduces nasal resistance, increases the cross-sectional area of the nasal passages, and may lead to significant improvement in nasal airflow and respiratory efficiency [40,41,42,43,44,45].

For this reason, RME potentially contributes to improved respiratory patterns and quality of life in pediatric patients.

These morpho-functional effects often facilitate the re-establishment of nasal breathing, particularly in children with habitual mouth breathing due to maxillary constriction or nasal airway obstruction [46,47,48,49,50].

Given these additional benefits, RME has attracted growing interest in interdisciplinary contexts [51,52,53,54,55,56,57,58,59,60,61,62,63].

### 1.2. Upper Airway Obstruction in Children

Upper airway obstruction in pediatric patients is a complex clinical condition, which may arise from both anatomical and functional impairments [64,65,66,67,68,69,70,71,72]. The most common causes include adeno-tonsillar hypertrophy, deviated nasal septum, chronic rhinosinusitis, allergic rhinitis, nasal polyps, and structural craniofacial anomalies, such as a retrognathic mandible or maxillary hypoplasia [73,74,75,76,77,78,79,80,81,82,83]. These factors can act alone or in combination to produce varying degrees of airway narrowing, often leading to compensatory mouth breathing, especially during sleep.

One key consequence of upper airway obstruction is the adoption of chronic oral breathing. It alters orofacial muscle balance and may contribute to dysmorphic craniofacial growth.

This adaptation can result in a vertically exaggerated facial pattern, a high-arched (ogival) palate, a narrow maxillary arch, and underdeveloped midface [84,85,86,87,88,89,90,91].

Upper airway obstruction can compromise systemic health and quality of life. Disrupted breathing patterns can impair oxygenation, reduce sleep quality, and impact cognitive performance, emotional regulation, and physical growth [92,93,94,95,96,97,98,99,100]. It may impair sleep, oxygenation, cognition, growth, and behavior.

The presence of such an obstruction is also associated with the development of sleep-disordered breathing syndromes, such as primary snoring and obstructive sleep apnea, particularly in susceptible children [101,102,103,104,105,106,107,108].

**Figure 2:**
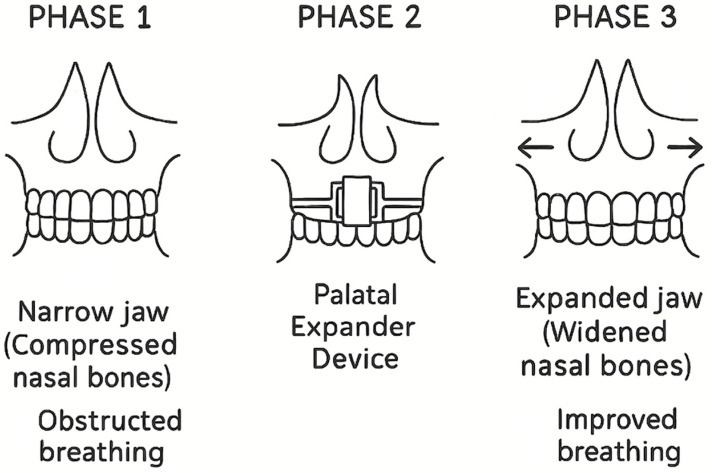
Increasing upper airway volume.

Given the risk of complications, early identification and treatment are crucial. A multidisciplinary approach involving pediatric, ENT, orthodontic, and sleep experts is recommended. Prompt intervention (Figure 2), whether medical, surgical, or orthopedic, may prevent more severe complications and support the optimal development of both respiratory and craniofacial functions [109,110,111,112,113,114,115,116].

### 1.3. Pediatric Obstructive Sleep Apnea (OSAS)

**Figure 3:**
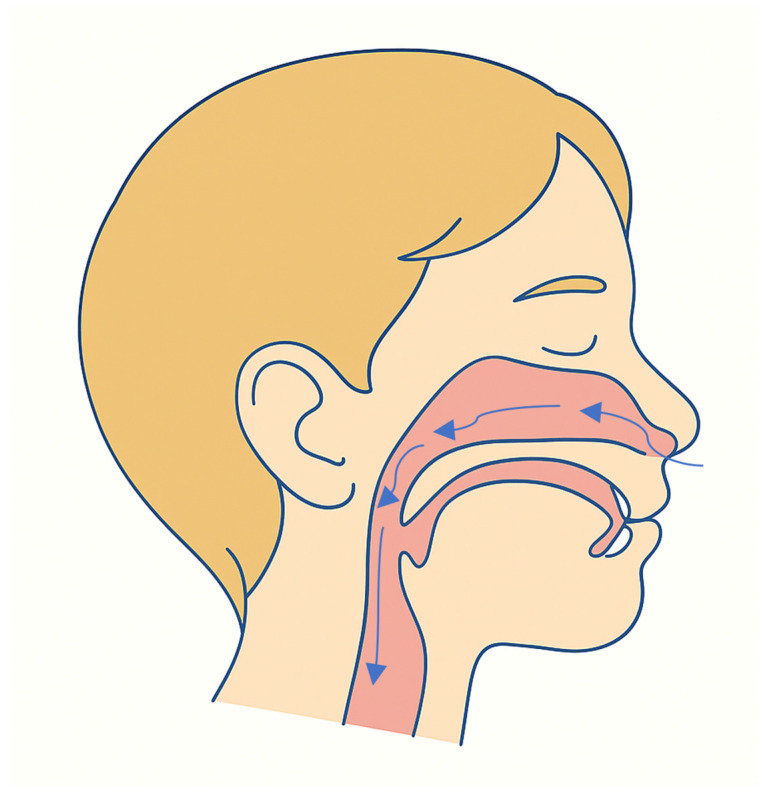
Sagittal section of the naso-oral region showing the respiratory airflow.

Obstructive sleep apnea syndrome (OSAS) in children is a prevalent condition, affecting approximately 1% to 5% of the pediatric population, depending on diagnostic criteria and population samples [117,118,119,120,121,122]. It is characterized by repetitive episodes of upper airway obstruction during sleep, which may be partial (hypopneas) or complete (apneas), leading to intermittent hypoxia, increased sympathetic nervous system activity, and repeated arousals that disturb the continuity and architecture of sleep (Figure 3) [123,124,125,126,127,128].

Sleep fragmentation may compromise neurocognitive development. Several studies have suggested that pediatric OSAS may be associated with behavioral changes, memory and attention deficits, learning difficulties, and poor academic achievement [129,130,131,132,133]. These symptoms may overlap with ADHD, increasing the risk of misdiagnosis.

Clinically, children with OSAS present a diverse constellation of signs and symptoms. Nocturnal features may include habitual snoring, labored or noisy breathing, gasping episodes, mouth breathing, frequent awakenings, and enuresis [134,135,136]. Daytime manifestations may involve excessive sleepiness or, paradoxically, hyperactivity, mood instability, headaches, difficulty concentrating, and poor school performance. Severe cases may show growth delays and an altered head posture [137,138].

Diagnosing pediatric OSAS requires a comprehensive and integrative approach. Clinical examination must be supplemented by evaluations from ENT specialists, orthodontists, and sleep experts. While questionnaires and overnight oximetry may provide preliminary information, polysomnography remains the gold standard for definitive diagnosis, allowing the quantification of apnea–hypopnea indices and sleep architecture disruptions [139,140,141,142]. Early recognition is essential to guide intervention and prevent complications.

### 1.4. Consequences and the Need for Early Intervention

If left untreated, pediatric OSAS may have far-reaching effects on physical, cognitive, and emotional development [143,144,145,146]. Research has consistently demonstrated strong associations between sleep-disordered breathing and negative health outcomes, including cognitive impairment, behavioral disturbances, emotional dysregulation, childhood obesity, insulin resistance, systemic hypertension, and delayed growth [147,148,149]. These effects are mediated by repeated oxygen desaturation, sleep fragmentation, and altered autonomic regulation, all of which impair the body’s ability to recover and develop during sleep.

Sleep disruption also affects endocrine functions involved in physical and cognitive maturation. Growth hormone, for instance, is secreted predominantly during deep sleep phases, and sleep disturbances may reduce its availability, impairing height gain and lean body mass development [150,151,152].

Due to the multifaceted impact of OSAS, early identification and prompt treatment are crucial clinical imperatives [153]. The most common first-line treatment for OSAS in children with adeno-tonsillar hypertrophy is adenotonsillectomy, which can significantly reduce airway obstruction and improve symptoms in many cases. However, in children with persistent symptoms, narrow maxillary arches, or craniofacial anomalies, complementary interventions such as orthodontic treatment—including RME—may be necessary [154,155].

An interdisciplinary strategy is essential, addressing anatomy, function, and growth. By addressing the root causes of airway obstruction and restoring functional balance, early orthopedic–orthodontic treatment can significantly improve both the health trajectory and the quality of life of pediatric patients.

## 2. Materials and Methods

### 2.1. Protocol and Registration

This systematic review was conducted according to Preferred Reporting Items for Systematic Reviews and Meta-Analyses (PRISMA). The review protocol was registered at The International Prospective Register of Systematic Reviews Registry guidelines (PROSPERO ID: 1063275).

### 2.2. Search Processing

A comprehensive literature search was conducted on PubMed, Scopus, and Web of Science to identify studies addressing the relationship between nasal breathing and maxillary expansion. The search covered the last 10 years (2015–2025) and was limited to publications in English. The search strategy was created by combining terms relevant to the study’s purpose. The following Boolean keywords were applied: “nasal” AND “breathing” AND “expansion” AND “maxillary”.

Due to heterogeneity among the included studies (differences in study design, patient populations, RME protocols, and outcomes), a formal meta-analysis was not performed. Data were synthesized qualitatively, and descriptive analyses were conducted using Microsoft Excel, version 2019 (Microsoft Corporation, Redmond, WA, USA), and IBM SPSS Statistics, version 28 (IBM Corp., Armonk, NY, USA), with a narrative comparison across studies to highlight trends and consistencies.

### 2.3. Inclusion and Exclusion Criteria

The studies included in this systematic review focused on children and adolescents with OSAS confirmed by polysomnography, defined according to international pediatric guidelines (ICSD-3) as recurrent episodes of apnea or hypopnea during sleep, with severity determined by the apnea–hypopnea index (AHI) > 1 event/hour in pediatric patients. In addition, the presence of mouth breathing, defined as the predominant passage of air through the oral cavity, was required, identified through clinical assessment or specific diagnostic tools reported in the original studies.

The following table (Table 1) outlines the inclusion and exclusion criteria adopted for the selection of studies in this systematic review. These criteria were defined to ensure the clinical relevance, appropriateness, and methodological quality of the included articles.

The review was conducted using the PICOS criteria (Table 2).

## 3. Results

**Figure 4:**
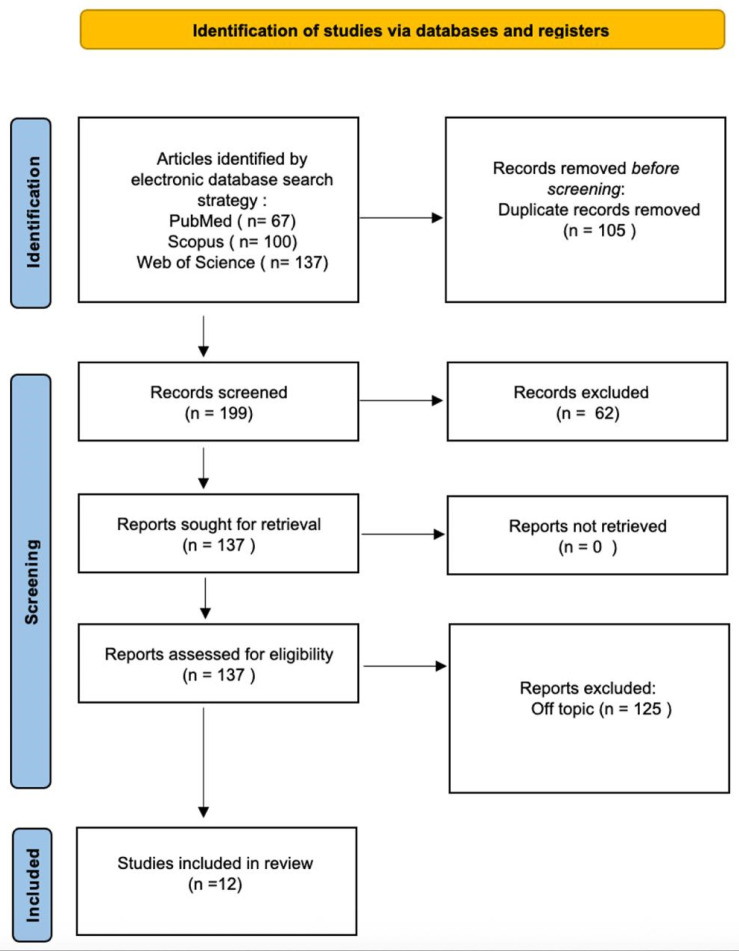
Literature search Preferred Reporting Items for Systematic Reviews and Meta-Analyses (PRISMA) flow diagram and database search indicators.

Three databases were searched, including 304 publications: Pubmed (67), Web of Science (137), and Scopus (100). After 105 duplicate entries were removed, 199 records were screened for titles and abstracts, leading to a further 62 articles being removed. Following a full-text review, 125 papers were excluded for failing to meet the inclusion criteria. A total of 12 publications (Table 3) were ultimately determined to be suitable for qualitative analysis (Table 2). The selection process is summarized in the PRISMA guidelines (Figure 4).

### 3.1. Data Processing

Three reviewers (M.C., L.P.Z., S.S.) independently consulted the databases to collect the studies and rated their quality based on selection criteria. The selected articles were downloaded in Zotero (Version 6.0.15). Any divergence between the four authors was settled by a discussion with one senior reviewer (F.I.).

### 3.2. Quality Assessment

Using ROBINS-I V2, a tool designed to assess the risk of bias in the results of non-randomized studies comparing the health effects of two or more interventions, three reviewers—M.C., L.P.Z., and S.S.—evaluated the quality of the included publications.

**Figure 5:**
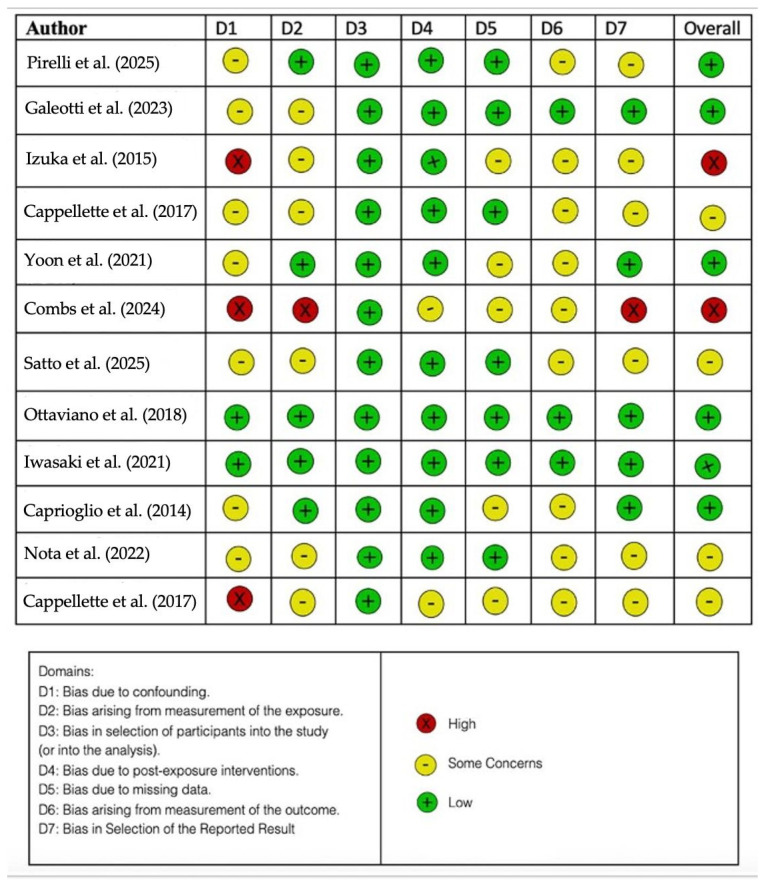
Bias assessment by ROBINS-I V2 [144,145,146,147,148,149,150,151,152,153,154,155].

Each of the seven assessed criteria was assigned a level of bias (Figure 5).

## 4. Discussion

This systematic review assessed the effectiveness of rapid maxillary expansion (RME) in improving both the structural and functional parameters of nasal respiration in pediatric patients with mouth breathing and/or sleep-disordered breathing (SDB), including obstructive sleep apnea (OSA). Despite methodological heterogeneity among the included studies, a consistent pattern emerges across the literature: RME leads to significant and measurable improvements in upper airway dimensions and nasal respiratory function, regardless of the diagnostic tools employed. The consistency of outcomes across diverse clinical scenarios and diagnostic modalities underscores the robust physiological impact of RME on the pediatric airway.

From a structural standpoint, several studies (Pirelli et al., 2025 [144]; Galeotti et al., 2023 [145]; Cappellette et al., 2017 [147]; Cappellette et al., 2017 [155]) using cone-beam computed tomography (CBCT) demonstrated volumetric increases in the nasal cavity, nasomaxillary complex, and posterior airway space following RME. These findings suggest that the mechanical separation of the midpalatal suture not only expands the maxillary arch but also induces favorable skeletal remodeling of the adjacent nasal and paranasal structures. The increase in nasal cavity dimensions translates into decreased nasal airway resistance, a factor directly implicated in improving nasal breathing. Such skeletal modifications have also been linked to the reestablishment of nasal breathing patterns in children previously reliant on oral breathing due to chronic obstruction.

These results are corroborated by studies employing other objective methods. For instance, Yoon et al. (2018) [148] used nasal endoscopy to demonstrate an increase in the internal nasal valve angle, a critical determinant of nasal airflow dynamics, along with a significant reduction in obstructive nasal symptoms. Ottaviano et al. (2018) [151] and Izuka et al. (2015) [146] employed rhinomanometry and quality-of-life questionnaires, respectively, both reporting significant improvements in nasal airflow and subjective respiratory comfort. These outcomes point to a convergence between radiographic, functional, and patient-reported improvements following RME, indicating a multifaceted therapeutic effect.

From a functional perspective, the included studies adopted various evaluation tools, yet they consistently reported enhancements in respiratory efficiency and breathing patterns. Iwasaki et al. (2021) [152] utilizing nasal airflow and capnographic assessments, observed improved nasal ventilation and a reduction in breathing effort post-RME. These physiological benefits are supported by Combs et al. (2024) [149] who, through spirometric measurements, reported sustained improvements in nasal airflow and pulmonary function during long-term follow-up. Such findings are particularly relevant in demonstrating that RME’s effects are not transient but may induce stable modifications in respiratory mechanics.

The study by Satto et al. (2025) [150] provides further long-term evidence, showing that RME promotes not only structural increases in airway volume but also a durable correction of mouth-breathing habits, maintained up to eight years post-treatment. These results support the hypothesis that RME can alter the functional trajectory of craniofacial and respiratory development during critical growth periods, thereby yielding lasting benefits in pediatric patients.

In children with OSA, the inclusion of polysomnographic data (Pirelli et al., 2025 [144]; Caprioglio et al., 2024 [153]) adds a higher level of diagnostic precision. These studies demonstrated significant reductions in the apnea–hypopnea index (AHI) and improvements in sleep architecture, which correlated positively with CBCT-measured increases in airway volume. This direct correspondence between anatomical expansion and functional respiratory outcomes reinforces the role of RME as a therapeutic tool not only in orthodontics but also in pediatric sleep medicine. Similarly, Nota et al. (2022) [154] reported cephalometric improvements in upper airway space and mandibular positioning, suggesting that RME may enhance craniofacial balance and contribute to airway patency, particularly during sleep.

While the included studies varied in design—ranging from randomized controlled trials (Yoon et al., 2021 [148]; Ottaviano et al., 2018 [151]; Iwasaki et al., 2021 [152]) to prospective (Combs et al., 2024 [149]; Caprioglio et al., 2014 [153]; Cappellette et al., 2017 [155]) and retrospective case–control designs (Galeotti et al., 2023 [145]; Nota et al., 2022 [154])—they all applied validated methodologies tailored to their objectives. Although the diagnostic tools differed (CBCT, PSG, rhinomanometry, nasal endoscopy, spirometry, and capnography), each method targeted a specific dimension of the nasal or respiratory system, and the convergence of their findings highlights the multifactorial improvements achieved through RME.

Importantly, the methodological variability across studies reflects the complexity of nasal breathing disorders and the need for multimodal assessment strategies. The use of both objective imaging and functional tests allowed for a comprehensive evaluation of RME’s impact, making the overall evidence more robust. Rather than undermining comparability, this heterogeneity enriches the evidence base by capturing diverse aspects of nasal airflow and airway function.

In conclusion, RME emerges as a highly effective, non-invasive intervention for pediatric patients with compromised nasal respiration and sleep-disordered breathing. Its efficacy lies in its dual impact: producing skeletal expansion that enhances anatomical airway patency and promoting functional respiratory adaptations that contribute to long-term health benefits. These findings support the integration of RME into interdisciplinary treatment strategies involving orthodontics, otolaryngology, and pediatric sleep medicine.

Despite the promising results, some limitations must be acknowledged. The included studies show variability in design, sample size, diagnostic tools, and outcome measures, which limits quantitative synthesis and generalizability. Future research should focus on larger, multicenter randomized trials with standardized protocols and long-term follow-up to confirm these findings and provide clearer clinical guidelines for the use of RME in pediatric patients with mouth breathing and sleep-disordered breathing.

## 5. Conclusions

The collective evidence presented in this systematic review supports the use of rapid maxillary expansion (RME) as an effective intervention for improving nasal airflow, upper airway volume, and sleep-related respiratory outcomes in children. Across diverse methodologies, all included studies documented structural and/or functional improvements following RME, regardless of whether patients were diagnosed with mouth breathing or obstructive sleep apnea.

Specifically, volumetric analyses via CBCT consistently demonstrated expansion of the nasomaxillary complex, with a consequent reduction in airway resistance. These changes were associated with improved nasal breathing, as confirmed by objective tools such as rhinomanometry and capnography, as well as subjective reports of improved respiratory comfort and sleep quality.

In OSA populations, RME was shown to significantly reduce AHI and enhance sleep architecture, as measured by polysomnography, thereby confirming its utility in functional airway rehabilitation. Additionally, long-term follow-up data (e.g., Satto et al., 2025 [150]) provide compelling evidence of the stability and durability of RME-induced improvements. Despite the lack of a single, unified methodology across studies, all employed clinically validated, objective diagnostic tools appropriate to their respective aims. This methodological diversity does not undermine the consistency of the findings; rather, it reflects the complex, multifactorial nature of upper airway obstruction and its assessment Although RME may contribute to improved airway patency, it should be considered as a complementary treatment to be implemented after addressing primary causes, such as adenotonsillar hypertrophy. In selected cases, RME can support functional recovery and contribute to an overall improvement in the quality of life of pediatric patients.

## Figures and Tables

**Table 1:** Inclusion and exclusion criteria.

Inclusion Criteria	Exclusion Criteria
Pediatric patients (<18 years) with OSAS or mouth breathing	Animal studies
Intervention with RME (Rapid Maxillary Expansion) using various types of expanders	Non-human or in vitro studies
Evaluation of outcomes: nasal resistance, nasal volume, airway patency, AHI, SpO_2_	Off-topic studies
Randomized clinical trials, prospective and case-control clinical trials	Reviews, retrospective studies, case series, case reports, letters to authors, or comments
Open-access studies written in English	Studies not written in English
Full-text articles	Studies without quantitative outcome data
Studies published in the last 10 years	

**Table 2:** Picos criteria.

PICO Element	Description
Population (P)	Children and adolescents (<18 years) diagnosed with obstructive sleep apnea syndrome (OSAS) or chronic mouth breathing. Participants typically presented with transverse maxillary constriction and/or nasal obstruction.
Intervention (I)	Treatment with rapid maxillary expansion (RME) using orthopedic or orthodontic appliances (e.g., Hyrax, Haas, bonded or banded expanders), activated at rates ranging from 0.25 to 0.5 mm per day. Diagnosis was confirmed through polysomnography (PSG) or based on clinical and symptomatic evaluation in combination with instrumental assessments such as CBCT, lateral cephalograms, and rhinomanometry.
Comparison (C)	Pre- and post-treatment assessments within the same group of patients. Some studies included control groups of untreated individuals or those receiving alternative therapies (e.g., pharmacological treatment, surgery, or CPAP in non-pediatric reference populations).
Outcome (O)	Reduction in apnea–hypopnea index (AHI); increase in nocturnal oxygen saturation; improvement in nasal airflow (assessed through rhinomanometry); increase in nasal cavity width and volume (measured via CBCT or cephalometry); improvement in subjective symptoms (e.g., snoring, nocturnal awakenings, daytime fatigue, sleep quality); long-term stability of results.

**Table 3:** Analysis of the study included in the discussion section [144,145,146,147,148,149,150,151,152,153,154,155].

Author (Year)	Type of Study	Aim	Material and Methods	Outcomes
Pirelli et al. (2025) Effect of rapid maxillary expansion on nasomaxillary structure and sleep disordered breathing in children with obstructive sleep apnoea [144]	Prospective clinical study	Improvement in OSAS and nasomaxillary structure	Children with OSAS treated with RME; evaluated AHI and nasomaxillary changes via CBCT	Significant reduction in AHI; increased nasomaxillary volume post-RME
Galeotti et al. (2023) Effects of Rapid Palatal Expansion on the Upper Airway Space in Children with Obstructive Sleep Apnea (OSA): A Case-Control Study [145]	Retrospective case–control	Increase in upper airway space	56 children (28 RME, 28 control); CBCT analysis pre- and post-expansion	Significant increase in nasopharyngeal and oropharyngeal volume in RME group
Izuka et al. (2015) Immediate impact of rapid maxillary expansion on upper airway dimensions and on the quality of life of mouth breathers [146]	Clinical trial	Airway changes and QOL improvement	25 children, CBCT and QOL questionnaires pre/post RME	Increased airway volume and improved subjective breathing scores
Cappellette et al. (2017) Impact of rapid maxillary expansion on nasomaxillary complex volume in mouth-breathers [147]	Clinical observational study	Nasomaxillary volume changes	Mouth-breathing children assessed with CBCT before and after RME	Significant increase in nasomaxillary complex volume
Yoon et al. (2021) Impact of rapid palatal expansion on the internal nasal valve and obstructive nasal symptoms in children [148]	Randomized controlled trial	Internal nasal valve area and nasal symptoms	Children with nasal obstruction; nasal endoscopy and symptom scoring pre/post RME	Increase in nasal valve angle and reduction in nasal obstruction symptoms
Combs et al. (2024) Long-term effects of maxillary skeletal expander treatment on functional breathing [149]	Longitudinal study	Functional breathing parameters	Long-term follow-up of children treated with skeletal RME; spirometry and clinical data	Sustained improvement in nasal airflow and respiratory function
Satto et al. (2025) Long-term structural and functional nasomaxillary evolution of children with mouth-breathing after rapid maxillary expansion: An 8-year follow-up study [150]	Long-term cohort study	Structural and functional evolution of nasomaxillary complex	Follow-up CBCT and clinical data after 8 years from RME in mouth-breathing children	Long-lasting increases in airway volume and correction of breathing pattern
Ottaviano et al. (2018) Nasal function before and after rapid maxillary expansion in children: A randomized, prospective, controlled study [151]	Randomized controlled trial	Nasal airflow resistance and breathing pattern	60 children randomized to RME or control; rhinomanometry and symptom questionnaires	RME significantly reduced nasal resistance and improved subjective nasal breathing
Iwasaki et al. (2021) Nasal ventilation and rapid maxillary expansion (RME): a randomized trial [152]	Randomized clinical trial	Nasal ventilation changes	Children with nasal obstruction randomized to RME or control; nasal airflow and capnography assessed	RME group showed increased nasal airflow and reduced breathing effort
Caprioglio et al. (2022) Rapid maxillary expansion in growing patients: Correspondence between 3-dimensional airway changes and polysomnography [153]	Prospective clinical study	Airway volume and OSAS severity	Growing patients with SDB assessed with CBCT and PSG pre/post RME	Increased airway volume correlated with improved polysomnographic parameters
Nota et al. (2022) Rapid Maxillary Expansion in Pediatric Patients with Sleep-Disordered Breathing: Cephalometric Variations in Upper Airway’s Dimension [154]	Retrospective case–control	Cephalometric airway changes	40 children (20 SDB, 20 control); cephalometric evaluation pre/post RME	Increase in airway dimensions and improved mandibular position
Cappellette et al. (2017) Skeletal effects of RME in the transverse and vertical dimensions of the nasal cavity in mouth-breathing growing children [155]	Prospective clinical study	Skeletal nasal cavity changes	25 children; CBCT pre/post RME to assess nasal cavity dimensions	Statistically significant increases in both transverse and vertical dimensions

## Data Availability

The data is contained within the article.

## Abbreviations

The following abbreviations are used in this manuscript:

AHI	apnea–hypopnea index
CBCT	cone-beam computed tomography
CIPAP	positive pressure airway devices
MAD	mandibular advancement devices
MRI	magnetic resonance imaging
OSA	obstructive sleep apnea
PSG	polysomnography
QOL	quality of life
SDB	sleep disordered breathing
UA	upper airway

## References

[1] Abate A., Lanteri V., Marcolongo L., Solimei L., Maspero C. (2023). Evaluation of Masticatory Muscles in Adult Patients with Maxillary Hypoplasia Treated with Surgically Assisted Rapid Maxillary Expansion (SARME): A Retrospective Study. J. Clin. Med..

[2] Badreddine F.R., Fujita R.R., Alves F.E.M.M., Cappellette M. (2018). Rapid Maxillary Expansion in Mouth Breathers: A Short-Term Skeletal and Soft-Tissue Effect on the Nose. Braz. J. Otorhinolaryngol..

[3] Balasubramanian S., Kalaskar R., Kalaskar A. (2022). Rapid Maxillary Expansion and Upper Airway Volume: Systematic Review and Meta-Analysis on the Role of Rapid Maxillary Expansion in Mouth Breathing. Int. J. Clin. Pediatr. Dent..

[4] Baratieri C.d.L., Alves M., Mattos C.T., Lau G.W.T., Nojima L.I., de Souza M.M.G. (2014). Transverse Effects on the Nasomaxillary Complex One Year after Rapid Maxillary Expansion as the Only Intervention: A Controlled Study. Dent. Press J. Orthod..

[5] Basciftci F.A., Mutlu N., Karaman A.I., Malkoc S., Küçükkolbasi H. (2002). Does the Timing and Method of Rapid Maxillary Expansion Have an Effect on the Changes in Nasal Dimensions?. Angle Orthod..

[6] Cerkezi S., Nakova M., Gorgoski I., Ferati K., Bexheti-Ferati A., Palermo A., Inchingolo A.D., Ferrante L., Inchingolo A.M., Inchingolo F. (2024). The Role of Sulfhydryl (Thiols) Groups in Oral and Periodontal Diseases. Biomedicines.

[7] Dipalma G., Inchingolo A.D., Memè L., Casamassima L., Carone C., Malcangi G., Inchingolo F., Palermo A., Inchingolo A.M. (2024). The Diagnosis and Management of Infraoccluded Deciduous Molars: A Systematic Review. Children.

[8] Dipalma G., Inchingolo A.M., Latini G., Ferrante L., Nardelli P., Malcangi G., Trilli I., Inchingolo F., Palermo A., Inchingolo A.D. (2024). The Effectiveness of Curcumin in Treating Oral Mucositis Related to Radiation and Chemotherapy: A Systematic Review. Antioxidants.

[9] Inchingolo A.D., Ferrara I., Viapiano F., Netti A., Campanelli M., Buongiorno S., Latini G., Carpentiere V., Ciocia A.M., Ceci S. (2022). Rapid Maxillary Expansion on the Adolescent Patient: Systematic Review and Case Report. Children.

[10] Inchingolo A.D., Inchingolo A.M., Malcangi G., Avantario P., Azzollini D., Buongiorno S., Viapiano F., Campanelli M., Ciocia A.M., De Leonardis N. (2022). Effects of Resveratrol, Curcumin and Quercetin Supplementation on Bone Metabolism—A Systematic Review. Nutrients.

[11] Langaliya A., Alam M.K., Hegde U., Panakaje M.S., Cervino G., Minervini G. (2023). Occurrence of Temporomandibular Disorders among Patients Undergoing Treatment for Obstructive Sleep Apnoea Syndrome (OSAS) Using Mandibular Advancement Device (MAD): A Systematic Review Conducted According to PRISMA Guidelines and the Cochrane Handbook for Systematic Reviews of Interventions. J. Oral Rehabil..

[12] Biederman W. (1968). A Hygienic Appliance for Rapid Expansion. JPO J. Pract. Orthod..

[13] Bishara S.E., Staley R.N. (1987). Maxillary Expansion: Clinical Implications. Am. J. Orthod. Dentofac. Orthop..

[14] Buck L.M., Dalci O., Darendeliler M.A., Papadopoulou A.K. (2016). Effect of Surgically Assisted Rapid Maxillary Expansion on Upper Airway Volume: A Systematic Review. J. Oral Maxillofac. Surg..

[15] Caldas L.D., Takeshita W.M., Machado A.W., Bittencourt M.A.V. (2020). Effect of Rapid Maxillary Expansion on Nasal Cavity Assessed with Cone-Beam Computed Tomography. Dent. Press J. Orthod..

[16] Cameron C.G., Franchi L., Baccetti T., McNamara J.A. (2002). Long-Term Effects of Rapid Maxillary Expansion: A Posteroanterior Cephalometric Evaluation. Am. J. Orthod. Dentofac. Orthop..

[17] Inchingolo F., Pacifici A., Gargari M., Acitores Garcia J.I., Amantea M., Marrelli M., Dipalma G., Inchingolo A.M., Rinaldi R., Inchingolo A.D. (2014). CHARGE Syndrome: An Overview on Dental and Maxillofacial Features. Eur. Rev. Med. Pharmacol. Sci..

[18] Inchingolo A.M., Patano A., De Santis M., Del Vecchio G., Ferrante L., Morolla R., Pezzolla C., Sardano R., Dongiovanni L., Inchingolo F. (2023). Comparison of Different Types of Palatal Expanders: Scoping Review. Children.

[19] Inchingolo A.M., Inchingolo A.D., Viapiano F., Ciocia A.M., Ferrara I., Netti A., Dipalma G., Palermo A., Inchingolo F. (2023). Treatment Approaches to Molar Incisor Hypomineralization: A Systematic Review. J. Clin. Med..

[20] Inchingolo A.M., Inchingolo A.D., Trilli I., Ferrante L., Di Noia A., de Ruvo E., Palermo A., Inchingolo F., Dipalma G. (2024). Orthopedic Devices for Skeletal Class III Malocclusion Treatment in Growing Patients: A Comparative Effectiveness Systematic Review. J. Clin. Med..

[21] Inchingolo A.M., Inchingolo A.D., Nardelli P., Latini G., Trilli I., Ferrante L., Malcangi G., Palermo A., Inchingolo F., Dipalma G. (2024). Stem Cells: Present Understanding and Prospects for Regenerative Dentistry. J. Funct. Biomater..

[22] Tepedino M., Iancu-Potrubacz M., Ciavarella D., Masedu F., Marchione L., Chimenti C. (2018). Expansion of Permanent First Molars with Rapid Maxillary Expansion Appliance Anchored on Primary Second Molars. J. Clin. Exp. Dent..

[23] Sabatucci A., Raffaeli F., Mastrovincenzo M., Luchetta A., Giannone A., Ciavarella D. (2015). Breathing Pattern and Head Posture: Changes in Craniocervical Angles. Minerva Stomatol..

[24] Dipalma G., Inchingolo A.D., Pezzolla C., Sardano R., Trilli I., Di Venere D., Corsalini M., Inchingolo F., Severino M., Palermo A. (2025). Head and Cervical Posture in Sagittal Skeletal Malocclusions: Insights from a Systematic Review. J. Clin. Med..

[25] Inchingolo F., Hazballa D., Inchingolo A.D., Malcangi G., Marinelli G., Mancini A., Maggiore M.E., Bordea I.R., Scarano A., Farronato M. (2022). Innovative Concepts and Recent Breakthrough for Engineered Graft and Constructs for Bone Regeneration: A Literature Systematic Review. Materials.

[26] Ciavarella D., Monsurrò A., Padricelli G., Battista G., Laino L., Perillo L. (2012). Unilateral Posterior Crossbite in Adolescents: Surface Electromyographic Evaluation. Eur. J. Paediatr. Dent..

[27] Cantarella D., Dominguez-Mompell R., Moschik C., Mallya S.M., Pan H.C., Alkahtani M.R., Elkenawy I., Moon W. (2018). Midfacial Changes in the Coronal Plane Induced by Microimplant-Supported Skeletal Expander, Studied with Cone-Beam Computed Tomography Images. Am. J. Orthod. Dentofac. Orthop..

[28] Cerritelli L., Hatzopoulos S., Catalano A., Bianchini C., Cammaroto G., Meccariello G., Iannella G., Vicini C., Pelucchi S., Skarzynski P.H. (2022). Rapid Maxillary Expansion (RME): An Otolaryngologic Perspective. J. Clin. Med..

[29] Chang Y., Koenig L.J., Pruszynski J.E., Bradley T.G., Bosio J.A., Liu D. (2013). Dimensional Changes of Upper Airway after Rapid Maxillary Expansion: A Prospective Cone-Beam Computed Tomography Study. Am. J. Orthod. Dentofac. Orthop..

[30] Ciambotti C., Ngan P., Durkee M., Kohli K., Kim H. (2001). A Comparison of Dental and Dentoalveolar Changes between Rapid Palatal Expansion and Nickel-Titanium Palatal Expansion Appliances. Am. J. Orthod. Dentofac. Orthop..

[31] Colak O., Paredes N.A., Elkenawy I., Torres M., Bui J., Jahangiri S., Moon W. (2020). Tomographic Assessment of Palatal Suture Opening Pattern and Pterygopalatine Suture Disarticulation in the Axial Plane after Midfacial Skeletal Expansion. Prog. Orthod..

[32] Cross D.L., McDonald J.P. (2000). Effect of Rapid Maxillary Expansion on Skeletal, Dental, and Nasal Structures: A Postero-Anterior Cephalometric Study. Eur. J. Orthod..

[33] Meme’ L., Bambini F., Dipalma G., Sampalmieri F., Laforgia A., Inchingolo A.D., Pennacchio B.F.P., Giorgio R.V., Corsalini M., Paduanelli G. (2024). The key role of the palatal expander in orthodontics. Eur. J. Musculoskelet. Dis..

[34] Inchingolo F., Tatullo M., Abenavoli F.M., Marrelli M., Inchingolo A.D., Inchingolo A.M., Dipalma G. (2011). Non-Hodgkin Lymphoma Affecting the Tongue: Unusual Intra-Oral Location. Head Neck Oncol..

[35] Limongelli L., Cascardi E., Capodiferro S., Favia G., Corsalini M., Tempesta A., Maiorano E. (2020). Multifocal Amelanotic Melanoma of the Hard Palate: A Challenging Case. Diagnostics.

[36] Montemurro N., Pierozzi E., Inchingolo A.M., Pahwa B., De Carlo A., Palermo A., Scarola R., Dipalma G., Corsalini M., Inchingolo A.D. (2023). New Biograft Solution, Growth Factors and Bone Regenerative Approaches in Neurosurgery, Dentistry, and Orthopedics: A Review. Eur. Rev. Med. Pharmacol. Sci..

[37] Kang J.-H., Kim H.J., Song S.I. (2022). Obstructive Sleep Apnea and Anatomical Structures of the Nasomaxillary Complex in Adolescents. PLoS ONE.

[38] Kikuchi M. (2005). Orthodontic Treatment in Children to Prevent Sleep-Disordered Breathing in Adulthood. Sleep Breath..

[39] Leiter J.C. (1996). Upper Airway Shape: Is It Important in the Pathogenesis of Obstructive Sleep Apnea?. Am. J. Respir. Crit. Care Med..

[40] Ng A.T., Qian J., Cistulli P.A. (2006). Oropharyngeal Collapse Predicts Treatment Response with Oral Appliance Therapy in Obstructive Sleep Apnea. Sleep.

[41] Punjabi N.M. (2008). The Epidemiology of Adult Obstructive Sleep Apnea. Proc. Am. Thorac. Soc..

[42] Svanborg E. (1994). Obstructive sleep apnea syndrome. Current literature on diagnostic methods. Lakartidningen.

[43] Tangugsorn V., Krogstad O., Espeland L., Lyberg T. (2000). Obstructive Sleep Apnoea: Multiple Comparisons of Cephalometric Variables of Obese and Non-Obese Patients. J. Craniomaxillofac. Surg..

[44] Tishler P.V., Larkin E.K., Schluchter M.D., Redline S. (2003). Incidence of Sleep-Disordered Breathing in an Urban Adult Population: The Relative Importance of Risk Factors in the Development of Sleep-Disordered Breathing. JAMA.

[45] Iwasaki T., Saitoh I., Takemoto Y., Inada E., Kanomi R., Hayasaki H., Yamasaki Y. (2012). Improvement of Nasal Airway Ventilation after Rapid Maxillary Expansion Evaluated with Computational Fluid Dynamics. Am. J. Orthod. Dentofac. Orthop..

[46] Iwasaki T., Saitoh I., Takemoto Y., Inada E., Kakuno E., Kanomi R., Hayasaki H., Yamasaki Y. (2013). Tongue Posture Improvement and Pharyngeal Airway Enlargement as Secondary Effects of Rapid Maxillary Expansion: A Cone-Beam Computed Tomography Study. Am. J. Orthod. Dentofac. Orthop..

[47] Jose J., Ell S.R. (2003). The Association of Subjective Nasal Patency with Peak Inspiratory Nasal Flow in a Large Healthy Population. Clin. Otolaryngol. Allied Sci..

[48] Kabalan O., Gordon J., Heo G., Lagravère M.O. (2015). Nasal Airway Changes in Bone-Borne and Tooth-Borne Rapid Maxillary Expansion Treatments. Int. Orthod..

[49] Kara M., Erdoğan H., Güçlü O., Sahin H., Dereköy F.S. (2016). Evaluation of Sleep Quality in Patients with Nasal Septal Deviation via the Pittsburgh Sleep Quality Index. J. Craniofac. Surg..

[50] Kiliç N., Oktay H. (2008). Effects of Rapid Maxillary Expansion on Nasal Breathing and Some Naso-Respiratory and Breathing Problems in Growing Children: A Literature Review. Int. J. Pediatr. Otorhinolaryngol..

[51] Variation in Symptoms of Sleep-Disordered Breathing with Race and Ethnicity: The Sleep Heart Health Study. https://pubmed.ncbi.nlm.nih.gov/12627736/.

[52] Sleep-Disordered Breathing. https://pubmed.ncbi.nlm.nih.gov/23099131/.

[53] Predictors of Sleep-Disordered Breathing in Community-Dwelling Adults: The Sleep Heart Health Study. https://pubmed.ncbi.nlm.nih.gov/11966340/.

[54] Mandibular Advancement Devices in 630 Men and Women with Obstructive Sleep Apnea and Snoring: Tolerability and Predictors of Treatment Success. https://pubmed.ncbi.nlm.nih.gov/15078734/.

[55] Heart Rate Responses to Autonomic Challenges in Obstructive Sleep Apnea. https://pubmed.ncbi.nlm.nih.gov/24194842/.

[56] Farronato G., Giannini L., Galbiati G., Maspero C. (2012). Comparison of the Dental and Skeletal Effects of Two Different Rapid Palatal Expansion Appliances for the Correction of the Maxillary Asymmetric Transverse Discrepancies. Minerva Stomatol..

[57] Farronato G., Maspero C., Esposito L., Briguglio E., Farronato D., Giannini L. (2011). Rapid Maxillary Expansion in Growing Patients. Hyrax versus Transverse Sagittal Maxillary Expander: A Cephalometric Investigation. Eur. J. Orthod..

[58] Farronato M., Cenzato N., Crispino R., Tartaglia F.C., Biagi R., Baldini B., Maspero C. (2024). Divergence between CBCT and Optical Scans for Soft Tissue Analysis and Cephalometry in Facial Imaging: A Cross-Sectional Study on Healthy Adults. Int. Orthod..

[59] Maspero C., Cenzato N., Inchingolo F., Cagetti M.G., Isola G., Sozzi D., Del Fabbro M., Tartaglia G.M. (2023). The Maxilla-Mandibular Discrepancies through Soft-Tissue References: Reliability and Validation of the Anteroposterior Measurement. Children.

[60] Lanteri V., Farronato M., Ugolini A., Cossellu G., Gaffuri F., Parisi F.M.R., Cavagnetto D., Abate A., Maspero C. (2020). Volumetric Changes in the Upper Airways after Rapid and Slow Maxillary Expansion in Growing Patients: A Case-Control Study. Materials.

[61] Lipan M.J., Most S.P. (2013). Development of a Severity Classification System for Subjective Nasal Obstruction. JAMA Facial Plast. Surg..

[62] Magnusson A., Bjerklin K., Nilsson P., Jönsson F., Marcusson A. (2011). Nasal Cavity Size, Airway Resistance, and Subjective Sensation after Surgically Assisted Rapid Maxillary Expansion: A Prospective Longitudinal Study. Am. J. Orthod. Dentofac. Orthop..

[63] Martos-Cobo E., Mayoral-Sanz P., Expósito-Delgado A.-J., Durán-Cantolla J. (2022). Effect of Rapid Maxillary Expansion on the Apnoea-Hypopnoea Index during Sleep in Children. Syst. Rev. J. Clin. Exp. Dent..

[64] McNamara J.A., Lione R., Franchi L., Angelieri F., Cevidanes L.H.S., Darendeliler M.A., Cozza P. (2015). The Role of Rapid Maxillary Expansion in the Promotion of Oral and General Health. Prog. Orthod..

[65] Melsen B. (1972). A Histological Study of the Influence of Sutural Morphology and Skeletal Maturation on Rapid Palatal Expansion in Children. Trans. Eur. Orthod. Soc..

[66] Moss J.P. (1968). Rapid Expansion of the Maxillary Arch. II. Indications for Rapid Expansion. JPO J. Pract. Orthod..

[67] Adult Sleep Apnea Syndromes. https://pubmed.ncbi.nlm.nih.gov/7653425/.

[68] A Randomized, Controlled Study of a Mandibular Advancement Splint for Obstructive Sleep Apnea. https://pubmed.ncbi.nlm.nih.gov/11371418/.

[69] A Four Year Follow-Up of Sleep and Respiratory Measures in Elementary School-Aged Children with Sleep Disordered Breathing. https://pubmed.ncbi.nlm.nih.gov/23499429/.

[70] Therapeutic and Adverse Effects of Lasers in Dentistry: A Systematic Review. https://www.mdpi.com/2304-6732/10/6/650.

[71] Rapid Palate Expansion’s Impact on Nasal Breathing: A Systematic Review. https://pubmed.ncbi.nlm.nih.gov/39954405/.

[72] Nieto F.J., Young T.B., Lind B.K., Shahar E., Samet J.M., Redline S., D’Agostino R.B., Newman A.B., Lebowitz M.D., Pickering T.G. (2000). Association of Sleep-Disordered Breathing, Sleep Apnea, and Hypertension in a Large Community-Based Study. Sleep Heart Health Study. JAMA.

[73] De Felippe N.L.O., Da Silveira A.C., Viana G., Kusnoto B., Smith B., Evans C.A. (2008). Relationship between Rapid Maxillary Expansion and Nasal Cavity Size and Airway Resistance: Short- and Long-Term Effects. Am. J. Orthod. Dentofac. Orthop..

[74] Palaisa J., Ngan P., Martin C., Razmus T. (2007). Use of Conventional Tomography to Evaluate Changes in the Nasal Cavity with Rapid Palatal Expansion. Am. J. Orthod. Dentofac. Orthop..

[75] Pereira-Filho V.A., Monnazzi M.S., Gabrielli M.A.C., Spin-Neto R., Watanabe E.R., Gimenez C.M.M., Santos-Pinto A., Gabrielli M.F.R. (2014). Volumetric Upper Airway Assessment in Patients with Transverse Maxillary Deficiency after Surgically Assisted Rapid Maxillary Expansion. Int. J. Oral Maxillofac. Surg..

[76] Phatouros A., Goonewardene M.S. (2008). Morphologic Changes of the Palate after Rapid Maxillary Expansion: A 3-Dimensional Computed Tomography Evaluation. Am. J. Orthod. Dentofac. Orthop..

[77] Saccomanno S., Di Tullio A., D’Alatri L., Grippaudo C. (2019). Proposal for a Myofunctional Therapy Protocol in Case of Altered Lingual Frenulum. A Pilot Study. Eur. J. Paediatr. Dent..

[78] Saccomanno S., Martini C., D’Alatri L., Farina S., Grippaudo C. (2018). A Specific Protocol of Myo-Functional Therapy in Children with Down Syndrome. A Pilot Study. Eur. J. Paediatr. Dent..

[79] Influence of Respiratory Pattern on Craniofacial Growth. https://pubmed.ncbi.nlm.nih.gov/6947703/.

[80] Nasal Involvement in Obstructive Sleep Apnea Syndrome. https://pubmed.ncbi.nlm.nih.gov/25548569/.

[81] Non-Surgical Treatment of Transverse Deficiency in Adults Using Microimplant-Assisted Rapid Palatal Expansion (MARPE). https://pubmed.ncbi.nlm.nih.gov/28444019/.

[82] Darsey D.M., English J.D., Kau C.H., Ellis R.K., Akyalcin S. (2012). Does Hyrax Expansion Therapy Affect Maxillary Sinus Volume? A Cone-Beam Computed Tomography Report. Imaging Sci. Dent..

[83] De Felippe N.L.O., Bhushan N., Da Silveira A.C., Viana G., Smith B. (2009). Long-Term Effects of Orthodontic Therapy on the Maxillary Dental Arch and Nasal Cavity. Am. J. Orthod. Dentofac. Orthop..

[84] Di Carlo G., Saccucci M., Ierardo G., Luzzi V., Occasi F., Zicari A.M., Duse M., Polimeni A. (2017). Rapid Maxillary Expansion and Upper Airway Morphology: A Systematic Review on the Role of Cone Beam Computed Tomography. Biomed. Res. Int..

[85] Kaditis A.G., Alonso Alvarez M.L., Boudewyns A., Alexopoulos E.I., Ersu R., Joosten K., Larramona H., Miano S., Narang I., Trang H. (2016). Obstructive Sleep Disordered Breathing in 2- to 18-Year-Old Children: Diagnosis and Management. Eur. Respir. J..

[86] Marcus C.L., Brooks L.J., Draper K.A., Gozal D., Halbower A.C., Jones J., Schechter M.S., Sheldon S.H., Spruyt K., Ward S.D. (2012). Diagnosis and Management of Childhood Obstructive Sleep Apnea Syndrome. Pediatrics.

[87] Bharadwaj R., Ravikumar A., Krishnaswamy N.R. (2011). Evaluation of Craniofacial Morphology in Patients with Obstructive Sleep Apnea Using Lateral Cephalometry and Dynamic MRI. Indian J. Dent. Res..

[88] Duan J., Xia W., Li X., Zhang F., Wang F., Chen M., Chen Q., Wang B., Li B. (2025). Airway Morphology, Hyoid Position, and Serum Inflammatory Markers of Obstructive Sleep Apnea in Children Treated with Modified Twin-Block Appliances. BMC Oral Health.

[89] Eichenberger M., Baumgartner S. (2014). The Impact of Rapid Palatal Expansion on Children’s General Health: A Literature Review. Eur. J. Paediatr. Dent..

[90] Elkenawy I., Fijany L., Colak O., Paredes N.A., Gargoum A., Abedini S., Cantarella D., Dominguez-Mompell R., Sfogliano L., Moon W. (2020). An Assessment of the Magnitude, Parallelism, and Asymmetry of Micro-Implant-Assisted Rapid Maxillary Expansion in Non-Growing Patients. Prog. Orthod..

[91] Fastuca R., Meneghel M., Zecca P.A., Mangano F., Antonello M., Nucera R., Caprioglio A. (2015). Multimodal Airway Evaluation in Growing Patients after Rapid Maxillary Expansion. Eur. J. Paediatr. Dent..

[92] Fastuca R., Perinetti G., Zecca P.A., Nucera R., Caprioglio A. (2015). Airway Compartments Volume and Oxygen Saturation Changes after Rapid Maxillary Expansion: A Longitudinal Correlation Study. Angle Orthod..

[93] Fastuca R., Zecca P.A., Caprioglio A. (2014). Role of Mandibular Displacement and Airway Size in Improving Breathing after Rapid Maxillary Expansion. Prog. Orthod..

[94] Franklin K.A., Lindberg E. (2015). Obstructive Sleep Apnea Is a Common Disorder in the Population—A Review on the Epidemiology of Sleep Apnea. J. Thorac. Dis..

[95] Grippaudo C., Pantanali F., Paolantonio E.G., Saulle R., Latorre G., Deli R. (2013). Orthodontic Treatment Timing in Growing Patients. Eur. J. Paediatr. Dent..

[96] Oliva B., Sferra S., Greco A.L., Valente F., Grippaudo C. (2018). Three-Dimensional Analysis of Dental Arch Forms in Italian Population. Prog. Orthod..

[97] El H., Palomo J.M. (2011). Airway Volume for Different Dentofacial Skeletal Patterns. Am. J. Orthod. Dentofac. Orthop..

[98] Garrett B.J., Caruso J.M., Rungcharassaeng K., Farrage J.R., Kim J.S., Taylor G.D. (2008). Skeletal Effects to the Maxilla after Rapid Maxillary Expansion Assessed with Cone-Beam Computed Tomography. Am. J. Orthod. Dentofac. Orthop..

[99] Georgiadis T., Angelopoulos C., Papadopoulos M.A., Kolokitha O.-E. (2023). Three-Dimensional Cone-Beam Computed Tomography Evaluation of Changes in Naso-Maxillary Complex Associated with Rapid Palatal Expansion. Diagnostics.

[100] Ghoneima A., AlBarakati S., Jiang F., Kula K., Wasfy T. (2015). Computational Fluid Dynamics Analysis of the Upper Airway after Rapid Maxillary Expansion: A Case Report. Prog. Orthod..

[101] Haas A.J. (1965). The treatment of maxillary deficiency by opening the midpalatal suture. Angle Orthod..

[102] Haas A.J. (1970). Palatal Expansion: Just the Beginning of Dentofacial Orthopedics. Am. J. Orthod..

[103] Habeeb M., Boucher N., Chung C.-H. (2013). Effects of Rapid Palatal Expansion on the Sagittal and Vertical Dimensions of the Maxilla: A Study on Cephalograms Derived from Cone-Beam Computed Tomography. Am. J. Orthod. Dentofac. Orthop..

[104] Haralambidis A., Ari-Demirkaya A., Acar A., Küçükkeleş N., Ateş M., Ozkaya S. (2009). Morphologic Changes of the Nasal Cavity Induced by Rapid Maxillary Expansion: A Study on 3-Dimensional Computed Tomography Models. Am. J. Orthod. Dentofac. Orthop..

[105] Sakai R.-H.-U.-S., de Assumpção M.-S., Ribeiro J.-D., Sakano E. (2021). Impact of Rapid Maxillary Expansion on Mouth-Breathing Children and Adolescents: A Systematic Review. J. Clin. Exp. Dent..

[106] Sayar G., Kılınç D.D. (2019). Rapid Maxillary Expansion Outcomes According to Midpalatal Suture Maturation Levels. Prog. Orthod..

[107] Seif-Eldin N.F., Elkordy S.A., Fayed M.S., Elbeialy A.R., Eid F.H. (2019). Transverse Skeletal Effects of Rapid Maxillary Expansion in Pre and Post Pubertal Subjects: A Systematic Review. Open Access Maced. J. Med. Sci..

[108] Smith T., Ghoneima A., Stewart K., Liu S., Eckert G., Halum S., Kula K. (2012). Three-Dimensional Computed Tomography Analysis of Airway Volume Changes after Rapid Maxillary Expansion. Am. J. Orthod. Dentofac. Orthop..

[109] Snodell S.F., Nanda R.S., Currier G.F. (1993). A Longitudinal Cephalometric Study of Transverse and Vertical Craniofacial Growth. Am. J. Orthod. Dentofac. Orthop..

[110] Steegman R.M., Renkema A.-M., Schoeman A., Kuijpers-Jagtman A.M., Ren Y. (2023). Volumetric Changes in the Upper Airway on CBCT after Dentofacial Orthopedic Interventions—A Systematic Review. Clin. Oral Investig..

[111] Rapone B., Inchingolo A.D., Trasarti S., Ferrara E., Qorri E., Mancini A., Montemurro N., Scarano A., Inchingolo A.M., Dipalma G. (2022). Long-Term Outcomes of Implants Placed in Maxillary Sinus Floor Augmentation with Porous Fluorohydroxyapatite (Algipore^®^ FRIOS^®^) in Comparison with Anorganic Bovine Bone (Bio-Oss^®^) and Platelet Rich Plasma (PRP): A Retrospective Study. J. Clin. Med..

[112] Patano A., Inchingolo A.M., Cardarelli F., Inchingolo A.D., Viapiano F., Giotta M., Bartolomeo N., Di Venere D., Malcangi G., Minetti E. (2023). Effects of Elastodontic Appliance on the Pharyngeal Airway Space in Class II Malocclusion. J. Clin. Med..

[113] Minetti E., Palermo A., Malcangi G., Inchingolo A.D., Mancini A., Dipalma G., Inchingolo F., Patano A., Inchingolo A.M. (2023). Dentin, Dentin Graft, and Bone Graft: Microscopic and Spectroscopic Analysis. J. Funct. Biomater..

[114] Malcangi G., Patano A., Palmieri G., Di Pede C., Latini G., Inchingolo A.D., Hazballa D., de Ruvo E., Garofoli G., Inchingolo F. (2023). Maxillary Sinus Augmentation Using Autologous Platelet Concentrates (Platelet-Rich Plasma, Platelet-Rich Fibrin, and Concentrated Growth Factor) Combined with Bone Graft: A Systematic Review. Cells.

[115] Malcangi G., Patano A., Ciocia A.M., Netti A., Viapiano F., Palumbo I., Trilli I., Guglielmo M., Inchingolo A.D., Dipalma G. (2023). Benefits of Natural Antioxidants on Oral Health. Antioxidants.

[116] Malcangi G., Inchingolo A.D., Trilli I., Ferrante L., Casamassima L., Nardelli P., Inchingolo F., Palermo A., Severino M., Inchingolo A.M. (2025). Recent Use of Hyaluronic Acid in Dental Medicine. Materials.

[117] Teixeira R.U.F., Zappelini C.E.M., Alves F.S., da Costa E.A. (2011). Peak Nasal Inspiratory Flow Evaluation as an Objective Method of Measuring Nasal Airflow. Braz. J. Otorhinolaryngol..

[118] Vinha P.P., Faria A.C., Xavier S.P., Christino M., de Mello-Filho F.V. (2016). Enlargement of the Pharynx Resulting from Surgically Assisted Rapid Maxillary Expansion. J. Oral Maxillofac. Surg..

[119] Warren D.W., Hershey H.G., Turvey T.A., Hinton V.A., Hairfield W.M. (1987). The Nasal Airway Following Maxillary Expansion. Am. J. Orthod. Dentofac. Orthop..

[120] Wriedt S., Kunkel M., Zentner A., Wahlmann U.W. (2001). Surgically Assisted Rapid Palatal Expansion. An Acoustic Rhinometric, Morphometric and Sonographic Investigation. J. Orofac. Orthop..

[121] Yilmaz B.S., Kucukkeles N. (2014). Skeletal, Soft Tissue, and Airway Changes Following the Alternate Maxillary Expansions and Constrictions Protocol. Angle Orthod..

[122] Yu T.-T., Li J., Liu D.-W. (2020). Seven-Year Follow-up of the Nonsurgical Expansion of Maxillary and Mandibular Arches in a Young Adult: A Case Report. World J. Clin. Cases.

[123] Zambon C.E., Ceccheti M.M., Utumi E.R., Pinna F.R., Machado G.G., Peres M.P.S.M., Voegels R.L. (2012). Orthodontic Measurements and Nasal Respiratory Function after Surgically Assisted Rapid Maxillary Expansion: An Acoustic Rhinometry and Rhinomanometry Study. Int. J. Oral. Maxillofac. Surg..

[124] Laforgia A., Inchingolo A.M., Inchingolo F., Sardano R., Trilli I., Di Noia A., Ferrante L., Palermo A., Inchingolo A.D., Dipalma G. (2025). Paediatric Dental Trauma: Insights from Epidemiological Studies and Management Recommendations. BMC Oral Health.

[125] Inchingolo F., Tatullo M., Pacifici A., Gargari M., Inchingolo A.D., Inchingolo A.M., Dipalma G., Marrelli M., Abenavoli F.M., Pacifici L. (2012). Use of Dermal-Fat Grafts in the Post-Oncological Reconstructive Surgery of Atrophies in the Zygomatic Region: Clinical Evaluations in the Patients Undergone to Previous Radiation Therapy. Head Face Med..

[126] Inchingolo F., Tatullo M., Abenavoli F.M., Inchingolo A.D., Inchingolo A.M., Dipalma G. (2010). Fish-Hook Injuries: A Risk for Fishermen. Head Face Med..

[127] Inchingolo F., Tatullo M., Abenavoli F.M., Marrelli M., Inchingolo A.D., Inchingolo A.M., Dipalma G. (2010). Comparison between Traditional Surgery, CO2 and Nd:Yag Laser Treatment for Generalized Gingival Hyperplasia in Sturge-Weber Syndrome: A Retrospective Study. J. Investig. Clin. Dent..

[128] Inchingolo F., Inchingolo A.M., Latini G., Palmieri G., Di Pede C., Trilli I., Ferrante L., Inchingolo A.D., Palermo A., Lorusso F. (2023). Application of Graphene Oxide in Oral Surgery: A Systematic Review. Materials.

[129] Inchingolo F., Inchingolo A.M., Avantario P., Settanni V., Fatone M.C., Piras F., Di Venere D., Inchingolo A.D., Palermo A., Dipalma G. (2023). The Effects of Periodontal Treatment on Rheumatoid Arthritis and of Anti-Rheumatic Drugs on Periodontitis: A Systematic Review. Int. J. Mol. Sci..

[130] Mortellaro C., Dall’Oca S., Lucchina A.G., Castiglia A., Farronato G., Fenini E., Marenzi G., Trosino O., Cafiero C., Sammartino G. (2008). Sublingual Ranula: A Closer Look to Its Surgical Management. J. Craniofac. Surg..

[131] Sammartino G., Marenzi G., Colella G., Califano L., Grivetto F., Mortellaro C. (2005). Autogenous Calvarial Bone Graft Harvest: Intraoperational Complications. J. Craniofac. Surg..

[132] Gasparro R., Qorri E., Valletta A., Masucci M., Sammartino P., Amato A., Marenzi G. (2018). Non-Transfusional Hemocomponents: From Biology to the Clinic-A Literature Review. Bioengineering.

[133] Sammartino G., Cerone V., Gasparro R., Riccitiello F., Trosino O. (2014). Multidisciplinary Approach to Fused Maxillary Central Incisors: A Case Report. J. Med. Case Rep..

[134] Comparison of Skeletal and Dental Changes Between 2-Point and 4-Point Rapid Palatal Expanders. https://pubmed.ncbi.nlm.nih.gov/12637904/.

[135] Diagnosis and Treatment of Transverse Maxillary Deficiency. https://pubmed.ncbi.nlm.nih.gov/9082002/.

[136] Differential Assessment of Skeletal, Alveolar, and Dental Components Induced by Microimplant-Supported Midfacial Skeletal Expander (MSE), Utilizing Novel Angular Measurements from the Fulcrum. https://pubmed.ncbi.nlm.nih.gov/32656601/.

[137] Does Rapid Maxillary Expansion Affect Nasopharyngeal Airway? A Prospective Cone Beam Computerised Tomography (CBCT) Based Study. https://pubmed.ncbi.nlm.nih.gov/26827275/.

[138] Vagiakis E., Kapsimalis F., Lagogianni I., Perraki H., Minaritzoglou A., Alexandropoulou K., Roussos C., Kryger M. (2006). Gender Differences on Polysomnographic Findings in Greek Subjects with Obstructive Sleep Apnea Syndrome. Sleep Med..

[139] Tufik S., Santos-Silva R., Taddei J.A., Bittencourt L.R.A. (2010). Obstructive Sleep Apnea Syndrome in the Sao Paulo Epidemiologic Sleep Study. Sleep Med..

[140] Trindade S.H.K., Trindade I.E.K., Silva A.S.C.d., Araújo B.M.A.M., Trindade-Suedam I.K., Sampaio-Teixeira A.C.M., Weber S.A.T. (2022). Are Reduced Internal Nasal Dimensions a Risk Factor for Obstructive Sleep Apnea Syndrome?. Braz. J. Otorhinolaryngol..

[141] Caggiano M., Gasparro R., D’Ambrosio F., Pisano M., Di Palo M.P., Contaldo M. (2022). Smoking Cessation on Periodontal and Peri-Implant Health Status: A Systematic Review. Dent. J..

[142] Canfora F., Calabria E., Cuocolo R., Ugga L., Buono G., Marenzi G., Gasparro R., Pecoraro G., Aria M., D’Aniello L. (2021). Burning Fog: Cognitive Impairment in Burning Mouth Syndrome. Front. Aging Neurosci..

[143] D’Esposito V., Lecce M., Marenzi G., Cabaro S., Ambrosio M.R., Sammartino G., Misso S., Migliaccio T., Liguoro P., Oriente F. (2020). Platelet-Rich Plasma Counteracts Detrimental Effect of High-Glucose Concentrations on Mesenchymal Stem Cells from Bichat Fat Pad. J. Tissue Eng. Regen. Med..

[144] Pirelli P., Fiaschetti V., Mampieri G., Condo’ R., Ubaldi N., Pachi F., Giancotti A. (2025). Effect of rapid maxillary expansion on nasomaxillary structure and sleep disordered breathing in children with obstructive sleep apnoea. Aust. Dent. J..

[145] Galeotti A., Gatto R., Caruso S., Piga S., Maldonato W., Sitzia E., Viarani V., Bompiani G., Aristei F., Marzo G. (2023). Effects of Rapid Palatal Expansion on the Upper Airway Space in Children with Obstructive Sleep Apnea (OSA): A Case-Control Study. Children.

[146] Izuka E.N., Feres M.F.N., Pignatari S.S.N. (2015). Immediate Impact of Rapid Maxillary Expansion on Upper Airway Dimensions and on the Quality of Life of Mouth Breathers. Dent. Press J. Orthod..

[147] Cappellette M., Alves F.E.M.M., Nagai L.H.Y., Fujita R.R., Pignatari S.S.N. (2017). Impact of Rapid Maxillary Expansion on Nasomaxillary Complex Volume in Mouth-Breathers. Dent. Press J. Orthod..

[148] Yoon A., Abdelwahab M., Liu S., Oh J., Suh H., Trieu M., Kang K., Silva D. (2021). Impact of Rapid Palatal Expansion on the Internal Nasal Valve and Obstructive Nasal Symptoms in Children. Sleep Breath..

[149] Combs A., Paredes N., Dominguez-Mompell R., Romero-Maroto M., Zhang B., Elkenawy I., Sfogliano L., Fijany L., Colak O., Wu B. (2024). Long-Term Effects of Maxillary Skeletal Expander Treatment on Functional Breathing. Korean J. Orthod..

[150] Satto R.H.U., Sakuma E.T.I., Ribeiro J.D., Sakano E. (2025). Long-Term Structural and Functional Nasomaxillary Evolution of Children with Mouth-Breathing after Rapid Maxillary Expansion: An 8-Year Follow-up Study. Korean J. Orthod..

[151] Ottaviano G., Maculan P., Borghetto G., Favero V., Galletti B., Savietto E., Scarpa B., Martini A., Stellini E., De Filippis C. (2018). Nasal Function before and after Rapid Maxillary Expansion in Children: A Randomized, Prospective, Controlled Study. Int. J. Pediatr. Otorhinolaryngol..

[152] Iwasaki T., Papageorgiou S.N., Yamasaki Y., Ali Darendeliler M., Papadopoulou A.K. (2021). Nasal Ventilation and Rapid Maxillary Expansion (RME): A Randomized Trial. Eur. J. Orthod..

[153] Caprioglio A., Meneghel M., Fastuca R., Zecca P.A., Nucera R., Nosetti L. (2014). Rapid Maxillary Expansion in Growing Patients: Correspondence between 3-Dimensional Airway Changes and Polysomnography. Int. J. Pediatr. Otorhinolaryngol..

[154] Nota A., Caruso S., Caruso S., Sciarra F.M., Marino A., Daher S., Pittari L., Gatto R., Tecco S. (2022). Rapid Maxillary Expansion in Pediatric Patients with Sleep-Disordered Breathing: Cephalometric Variations in Upper Airway’s Dimension. Appl. Sci..

[155] Cappellette M., Nagai L.H.Y., Gonçalves R.M., Yuki A.K., Pignatari S.S.N., Fujita R.R. (2017). Skeletal Effects of RME in the Transverse and Vertical Dimensions of the Nasal Cavity in Mouth-Breathing Growing Children. Dent. Press J. Orthod..

